# The Effects of Xanthine Oxidoreductase Inhibitors on Oxidative Stress Markers following Global Brain Ischemia Reperfusion Injury in C57BL/6 Mice

**DOI:** 10.1371/journal.pone.0133980

**Published:** 2015-07-31

**Authors:** Masahiro Yamaguchi, Ken Okamoto, Teruo Kusano, Yoko Matsuda, Go Suzuki, Akira Fuse, Hiroyuki Yokota

**Affiliations:** 1 Nippon Medical School, Emergency and Critical Care Medicine, Tokyo, Japan; 2 Nippon Medical School, Biochemistry and Molecular Biology, Tokyo, Japan; 3 Tokyo Metropolitan Geriatric Hospital and Institute of Gerontology, Department of Pathology, Tokyo, Japan; National University of Singapore, SINGAPORE

## Abstract

We demonstrated that 3-nitrotyrosine and 4-hydroxy-2-nonenal levels in mouse brain were elevated from 1 h until 8 h after global brain ischemia for 14 min induced with the 3-vessel occlusion model; this result indicates that ischemia reperfusion injury generated oxidative stress. Reactive oxygen species production was observed not only in the hippocampal region, but also in the cortical region. We further evaluated the neuroprotective effect of xanthine oxidoreductase inhibitors in the mouse 3-vessel occlusion model by analyzing changes in the expression of genes regulated by the transcription factor nuclear factor-kappa B (including pro-inflammatory cytokines interleukin-1β (IL-1β) and tumor necrosis factor-α (TNF-α), matrix metalloproteinase-9 and intercellular adhesion molecules-1). Administration of allopurinol resulted in a statistically significant decrease in IL-1β and TNF-α mRNA expression, whereas febuxostat had no significant effect on expression of these genes; nevertheless, both inhibitors effectively reduced serum uric acid concentration. It is suggested that the neuroprotective effect of allopurinol is derived not from inhibition of reactive oxygen species production by xanthine oxidoreductase, but rather from a direct free-radical-scavenging effect.

## Introduction

Brain injury caused by global cerebral ischemia following cardiopulmonary arrest often results in severe clinical conditions, such as post cardiac arrest syndrome. A plausible explanation for the neuronal damage is that oxidative stress resulting from the generation of reactive oxygen species (ROS), including superoxide, hydrogen peroxide, and peroxynitrite,[1] occurs during the course of brain ischemia reperfusion (I/R). It has been demonstrated that ROS are directly involved in the oxidative damage to cellular macromolecules, such as proteins, lipids, and nucleic acids, in ischemic tissues, leading to cell death. However, the involvement of ROS in whole brain ischemia and I/R damage is still not well studied. Because of the limitations of genetically modified animals, many mouse models of global cerebral ischemia have been developed. A simple method of bilateral common carotid artery occlusion is most frequently used in mice.[2] However, this 2-vessel occlusion model failed to produce consistent histological brain damage, because mice have inter-individual differences in the collateral flow through the circle of Willis.[2] The 3-vessel occlusion model leverages combined occlusions of the basilar artery and both carotid arteries. This model produces satisfactory ischemia with cortical regional cerebral blood flow that is consistently below 10% of the baseline.[3]

Xanthine oxidoreductase (XOR) catalyzes the oxidation of hypoxanthine to xanthine and xanthine to uric acid, and the reduction of NAD^+^ or molecular oxygen. Mammalian XOR exists as xanthine dehydrogenase (XDH) in most tissues and prefers NAD^+^ as an electron donor. However, XDH is converted to xanthine oxidase (XO) in some situations, and XO reduces O_2_ to generate O_2_^-^ and H_2_O_2_. There have been many reports showing that ROS are generated by XO during cerebral I/R injury.[4, 5] XO inhibitors inhibit the conversion of xanthine to uric acid and are thus used as anti-gout drugs to suppress the toxic overproduction of ROS. Allopurinol and febuxostat are widely used inhibitors for treating gout and hyperuricemia. We previously used the 3-vessel occlusion model to perform a pathological evaluation of the effects of XOR inhibitors in the CA1 and CA2 regions of the hippocampus at 4 days after I/R, and found that allopurinol and febuxostat did not decrease brain I/R damage in mice.[6]

In this study, we further observed the generation of ROS in the 3-vessel occlusion model, and we examined whether XO is the major source of ROS in the I/R mouse brain.

## Methods

### Animal preparation

Male C57BL/6 (CLEA Japan Inc., Tokyo, Japan) mice aged 6 to 9 weeks were used in this study. All experimental animal procedures were approved by the institutional animal care committee of Nippon Medical School (Permit Number: 26–083). Efforts were made to minimize suffering and to minimize the number of animals used.

### Drug administration

Febuxostat, 2-[3-cyano-4-(2-methylpropoxy)phenyl]-4-methyl-5-thiazolecarboxylic acid, was obtained from Carbosynth Ltd. (Berkshire, UK). Allopurinol [4-hydroxypyrazolo(3,4-d)pyrimidine] was obtained from Sigma-Aldrich Co. LLC (St. Louis, MO, USA). The mice were administered the XOR inhibitors (febuxostat, allopurinol) orally at 50 mg/kg 30 min prior to the start of surgery; the same volume of 0.5% methylcellulose was administered orally to the placebo group.

### Surgical procedure

A global cerebral ischemia model was prepared as described.[3, 6] Briefly, after induction, anesthesia was maintained with 2.0% halothane in room air, delivered via a facemask. After a midline cervical incision, the bilateral common carotid arteries and the basilar artery were isolated. The basilar artery was occluded with a 0.2 mm diameter vascular clip (Fujita Medical Instruments Co., Ltd., Tokyo, Japan). Both common carotid arteries were occluded using 2 Sugita temporary miniclips (Mizuho Ikakogyo Co., Ltd., Tokyo, Japan). After 14 min of ischemia, the 3 clips were removed. The rectal temperature was maintained at 36.0°C–37.0°C with a heating blanket (Animal Blanket Controller, Model ATB-1100, Nihon Kohden Corporation, Tokyo, Japan). We confirmed that mice did not exhibit severe hypertension or hypotension after this procedure (n = 2). Control mice did not receive the operation. The mice were anesthetized with an intraperitoneal administration of pentobarbital (50 mg/kg) 1, 2, 4, 6, 8, and 96 h after I/R injury (n = 7). For western blot and PCR analyses, the mice were transcardially perfused with heparin containing normal saline, and the brains were frozen at -80°C. For histopathological analyses, the mice were transcardially perfused with 10% neutral buffered formalin, and the brains were removed and fixed overnight.

### Western blot analysis

We followed the method used in our previous report with minor modifications.[6] The tissue samples were homogenized with 50 mM potassium phosphate buffer (pH 7.4), 0.25 M sucrose, 1 mM salicylate, 0.3 mM EDTA, a Pierce Phosphatase Inhibitor Mini Tablet (Thermo Fisher Scientific Inc., Rockford, IL, USA), a complete protease inhibitor cocktail (Roche Applied Science, Indianapolis, IN, USA), and 1 mM dithiothreitol. The total protein concentration was determined using a Coomassie (Bradford) Protein Assay Reagent (Thermo Fisher Scientific Inc., Rockford, IL, USA). The samples were heated to 60°C for 3 min with Laemmli’s sample buffer, because 3-nitrotyrosine is easily reduced to aminotyrosine by heating in 2-mercaptoethanol-containing Laemmli’s sample buffer.[7] Equal amounts of protein were resolved with sodium dodecyl sulfate-polyacrylamide gel electrophoresis (Mini-PROTEAN TGX 4%–20%, Bio-Rad Laboratories, Inc., Hercules, CA, USA), and bands were electrophoretically transferred to a polyvinylidene fluoride membrane.

For blocking, antibody incubation, and membrane washing and reactions, we followed the protocols described in detail in a previous paper.[6] We used anti-3-nitrotyrosine antibody (1:1000, Santa Cruz Biotechnology, Inc., CA, USA), anti-4-hydroxy-2-nonenal (4-HNE) antibody (1:1000, JaICA Shizuoka, Japan), or anti-β-actin (1:1,000; BioVision, CA, USA) as the primary antibody, and a horseradish peroxidase-conjugated anti-mouse or anti-rabbit immunoglobulin antibody (1:1000 Dako Denmark A/S, Glostrup, Denmark) as the secondary antibody.

Immunoreactive bands were visualized and analyzed with the ChemiDoc XRS Plus System with Image Lab SVP Software (Bio-Rad Laboratories, Inc.).

### Histopathological analysis

The fixed tissues were cut into 2-mm thick coronal sections using a brain slicer (Muromachi Kikai Co., Ltd., Tokyo, Japan). The paraffin-embedded sections (3-μm thickness) were subjected to immunohistochemical staining. Tissue sections were incubated with anti-3-nitrotyrosine antibody (diluted at 1:25, LifeSpan BioSciences, Inc., Washington, USA), anti-4-HNE antibody (diluted at 1:50, JaICA Shizuoka, Japan), and anti-nuclear factor-kappa B (NF-κB) antibody (diluted at 1:100, Santa Cruz Biotechnology, Inc., CA, USA). Bound antibodies were detected using diaminobenzidine tetrahydrochloride as the substrate. The sections were then counterstained with Mayer's hematoxylin and reviewed by a pathologist blinded as to the source of the specimens. The CA1 and CA2 regions of the hippocampus and motor cortex in both hemispheres were evaluated for expression of 3-nitrotyrosine, 4-HNE and anti-NF-κB.

### Real-time PCR

The tissue samples were homogenized in RNAiso Plus reagent (Takara Bio Inc., Shiga, Japan). Total RNA was extracted from the tissue according to the manufacturer’s protocol. The RNA concentration was determined using a NanoDrop 2000 (Thermo Scientific, Delaware, ME, USA). Complementary DNA synthesis was performed using 5×PrimeScript Buffer, PrimeScript RT Enzyme Mix I, Oligo dT Primer, Random 6mers (Takara, Shiga, Japan), and total RNA. Real-time PCR and data analysis were performed using the ABI 7300 sequence detection system (Applied Biosystems, Foster City, CA). SYBR Premix Ex Taq II (Tli RNaseH Plus) and ROX Reference Dye were purchased from Takara Bio Inc., Shiga, Japan. Primer sequences are listed in Table 1. The PCR program was 30 sec at 95°C, then 40 cycles of denaturation at 95°C for 5 sec and annealing at 60°C for 31 sec, with a 5-min final extension period at 72°C. The amounts of interleukin-1β (IL-1β), tumor necrosis factor-α (TNFα), intercellular adhesion molecule-1 (ICAM-1), matrix metalloproteinase-9 (MMP-9), and xanthine dehydrogenase (Xdh) mRNA were normalized to β-actin expression using the threshold cycle method.

**Table 1:** Sequences of primers.

Gene	Primer	Sequence
*IL-1β*	Forward	5'-TCCAGGATGAGGACATGAGCAC-3'
	Reverse	5'-GAACGTCACACACCAGCAGGTTA-3'
*TNF-α*	Forward	5'-TATGGCCCAGACCCTCACA-3'
	Reverse	5'-GGAGTAGACAAGGTACAACCCATC-3'
*ICAM-1*	Forward	5'-CATGCCGCACAGAACTGGA-3'
	Reverse	5'-AGCTTTGGGATGGTAGCTGGAA-3'
*MMP-9*	Forward	5'-GCCCTGGAACTCACACGACA-3'
	Reverse	5'-TTGGAAACTCACACGCCAGAAG-3'
*Xdh*	Forward	5'-CAGCTTTGAGACAAACTCTGGGAAC-3'
	Reverse	5'-AAGCTGGAACCAACATCCATGAC-3'
*β-actin*	Forward	5'-CATCCGTAAAGACCTCTATGCCAAC-3'
	Reverse	5'-ATGGAGCCACCGATCCACA-3'

IL1β; interleukin-1β, TNF-α; tumor necrosis factor-α, ICAM-1; intercellular adhesion molecule-1, MMP-9; matrix metalloproteinase-9, Xdh; xanthine dehydrogenase.

### Statistical Analysis

Data were expressed as the mean ± S.D. and were analyzed for significance using repeated-measures ANOVA with a Greenhouse-Geisser correction followed by post hoc tests using the Bonferroni correction and one-way ANOVA, as appropriate. p<0.05 was considered statistically significant.

## Results

### Semi-quantitative post I/R time course of protein nitration and lipid peroxidation

**Fig 1:**
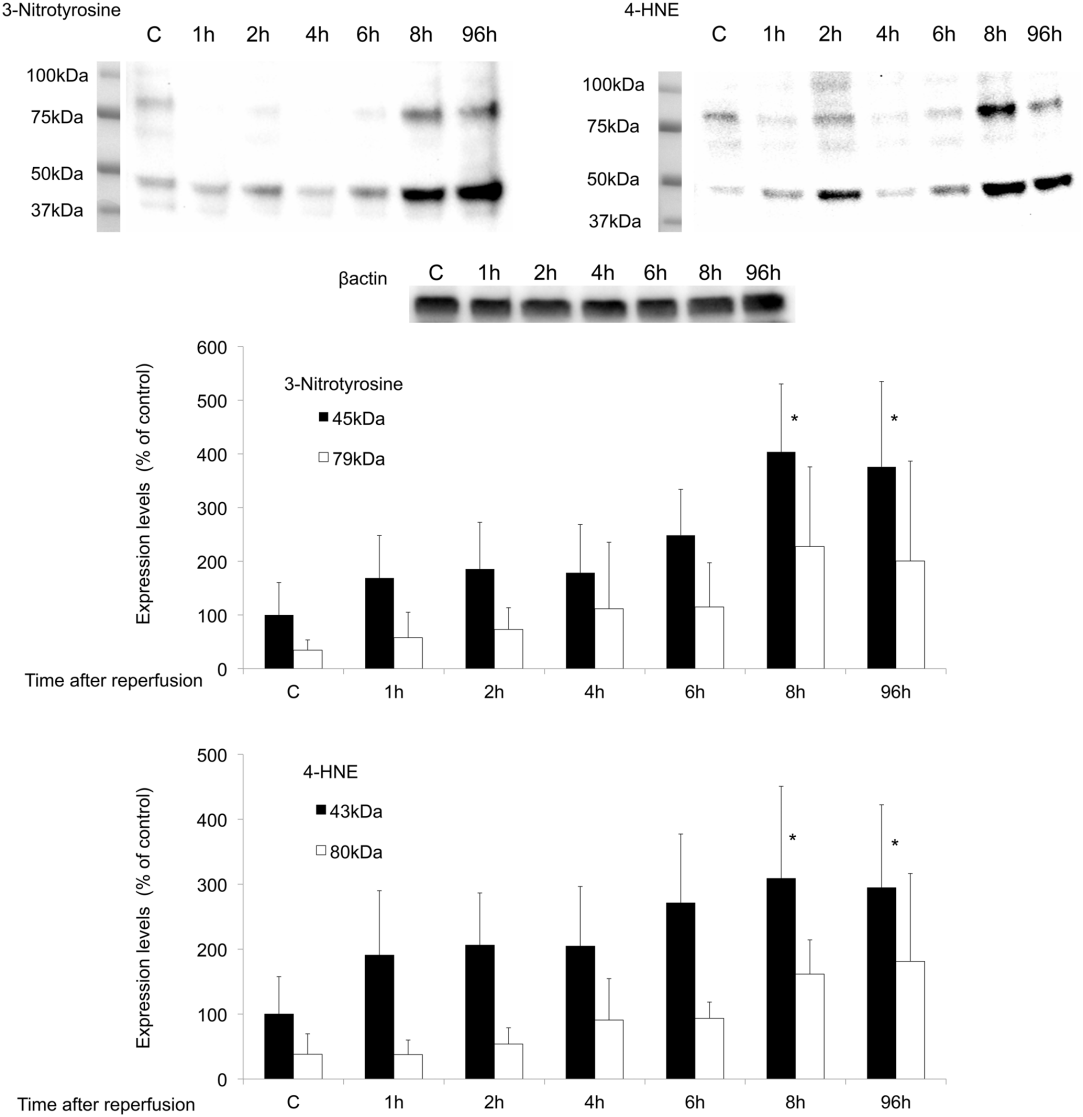
Time course of western blotting studies for monitoring protein nitration and lipid peroxidation. Solid bars represent 3-nitrotyrosine (45 kDa) and 4-HNE (43 kDa) values. Open bars represent 3-nitrotyrosine (79 kDa) and 4-HNE (80 kDa) values. The intensity levels are given as the mean ± SD normalized to the β-actin level for each sample (n = 7). Data were analyzed for significance using repeated-measures ANOVA with a Greenhouse-Geisser correction followed by post hoc tests using the Bonferroni correction. *p<0.05 vs. control. The entire images are shown in S1–S3 Figs.

Fig 1 show the results of a quantitative time course study of 3-nitrotyrosine- and 4-HNE-modified proteins measured by western blotting in samples taken from control (non-operated mice) or I/R injured mice. I/R injury produced a progressive increase in the levels of 3-nitrotyrosine and 4-HNE. Western blotting with anti-3-nitrotyrosine antibody showed immunoreactive protein of approximately 45 kDa and 79 kDa, and anti-4-HNE antibody showed immunoreactive protein of approximately 43 kDa and 80 kDa. The relative intensity of the staining versus β-actin is shown in Fig 1. Both the 3-nitrotyrosine and 4-HNE levels increased from 1 h to 96 h after reperfusion. Repeated-measures ANOVA with a Greenhouse-Geisser correction showed that the intensities of the 3-nitrotyrosine- (45 kDa) and 4-HNE- (43 kDa) positive signals differed statistically significantly between time points (3-nitrotyrosine p<0.001, 4-HNE p<0.01). Post hoc tests using the Bonferroni correction revealed that the intensities of the 3-nitrotyrosine- (45 kDa) and 4-HNE- (43 kDa) positive signals were significantly higher than those in the control at 8 h (3-nitrotyrosine p = 0.032, 4-HNE p = 0.033) and 96 h (3-nitrotyrosine p = 0.048, 4-HNE p = 0.049). The intensities of the 3-nitrotyrosine (79 kDa) and 4-HNE (80 kDa) bands were not statistically significant.

### Immunohistochemical analysis

**Fig 2:**
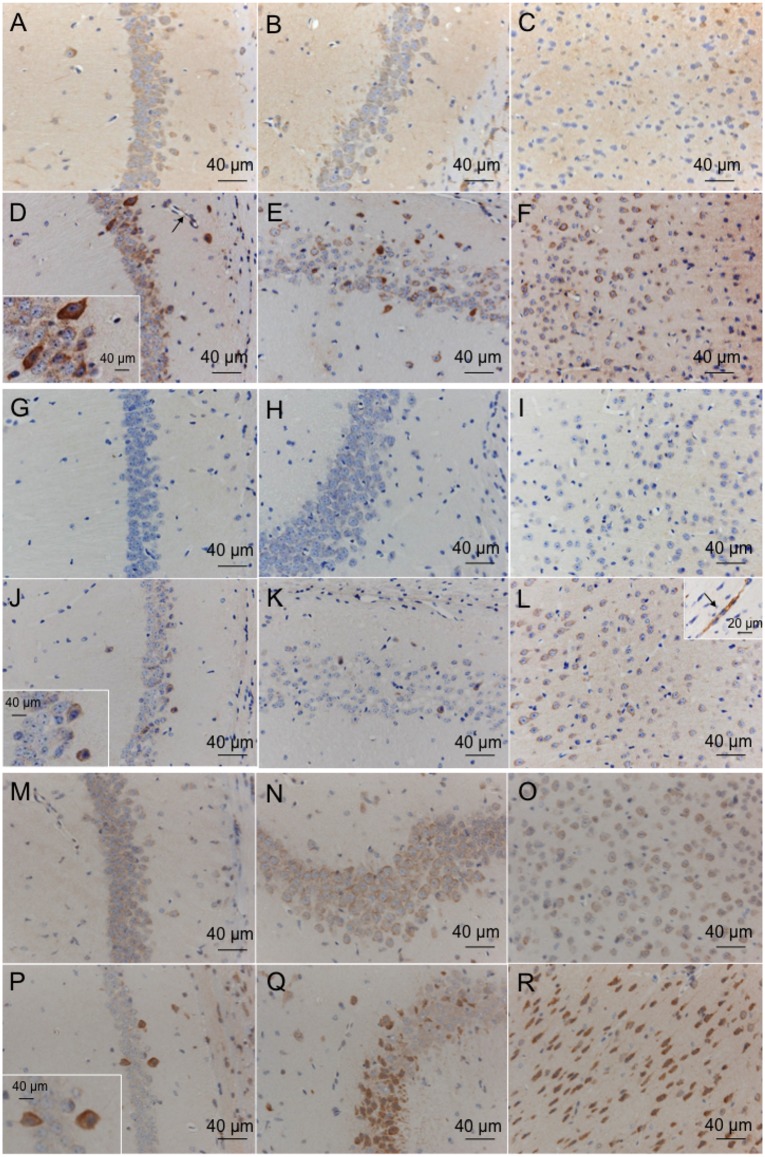
Distribution of immunoreactivity for 3-nitrotyrosine and 4-HNE in mice after global brain I/R injury. Sections were prepared from the CA1 (3-nitrotyrosine, A; 4-HNE, G; NF-κB, M), CA2 (3-nitrotyrosine, B; 4-HNE, H; NF-κB, N), and motor cortex (3-nitrotyrosine, C; 4-HNE, I; NF-κB, O) regions of the control brain and after 14 min of ischemia and 8 h of reperfusion in the CA1 (3-nitrotyrosine, D; 4-HNE, J; NF-κB, P), CA2 (3-nitrotyrosine, E; 4-HNE, K; NF-κB, Q) and motor cortex (3-nitrotyrosine, F; 4-HNE, L; NF-κB, R) regions of the brain. Arrow indicates vascular endothelium.

In control mice, the expression of 3-nitrotyrosine, 4-HNE and NF-κB was weak in the neurons of CA1 (3-nitrotyrosine; Fig 2A, 4-HNE; Fig 2G, NF-κB; Fig 2M), CA2 (3-nitrotyrosine; Fig 2B, 4-HNE; Fig 2H, NF-κB; Fig 2N) and the motor cortex (3-nitrotyrosine; Fig 2C, 4-HNE; Fig 2I, NF-κB; Fig 2O). At 8 h after I/R injury, several neurons showed strong expression of 3-nitrotyrosine (Fig 2D, CA1; Fig 2E, CA2; and Fig 2F, motor cortex), 4-HNE (Fig 2J, CA1; Fig 2K, CA2; and Fig 2L, motor cortex) and NF-κB (Fig 2P, CA1; Fig 2Q, CA2; and Fig 2R, motor cortex,) in vehicle-treated I/R mice. The vascular endothelium showed strong expression of 3-nitrotyrosine (Fig 2D, CA1; arrow indicates vascular endothelium) and 4-HNE (Fig 2L, motor cortex; arrow indicates vascular endothelium) in vehicle-treated I/R mice. At 24 and 96 h after I/R injury, the expression levels of 3-nitrotyrosine, 4-HNE and NF-κB were similar to those at 8 h after I/R injury.

### The effects of XOR inhibitors on 3-nitrotyrosine and 4-HNE levels in global brain I/R injury

The time course measured by means of western blot analysis showed maximum levels of 3-nitrotyrosine and 4-HNE at 8 h, and these levels did not change until 96 h (Fig 1). Thus, drug studies were performed after 24 h of reperfusion.

**Fig 3:**
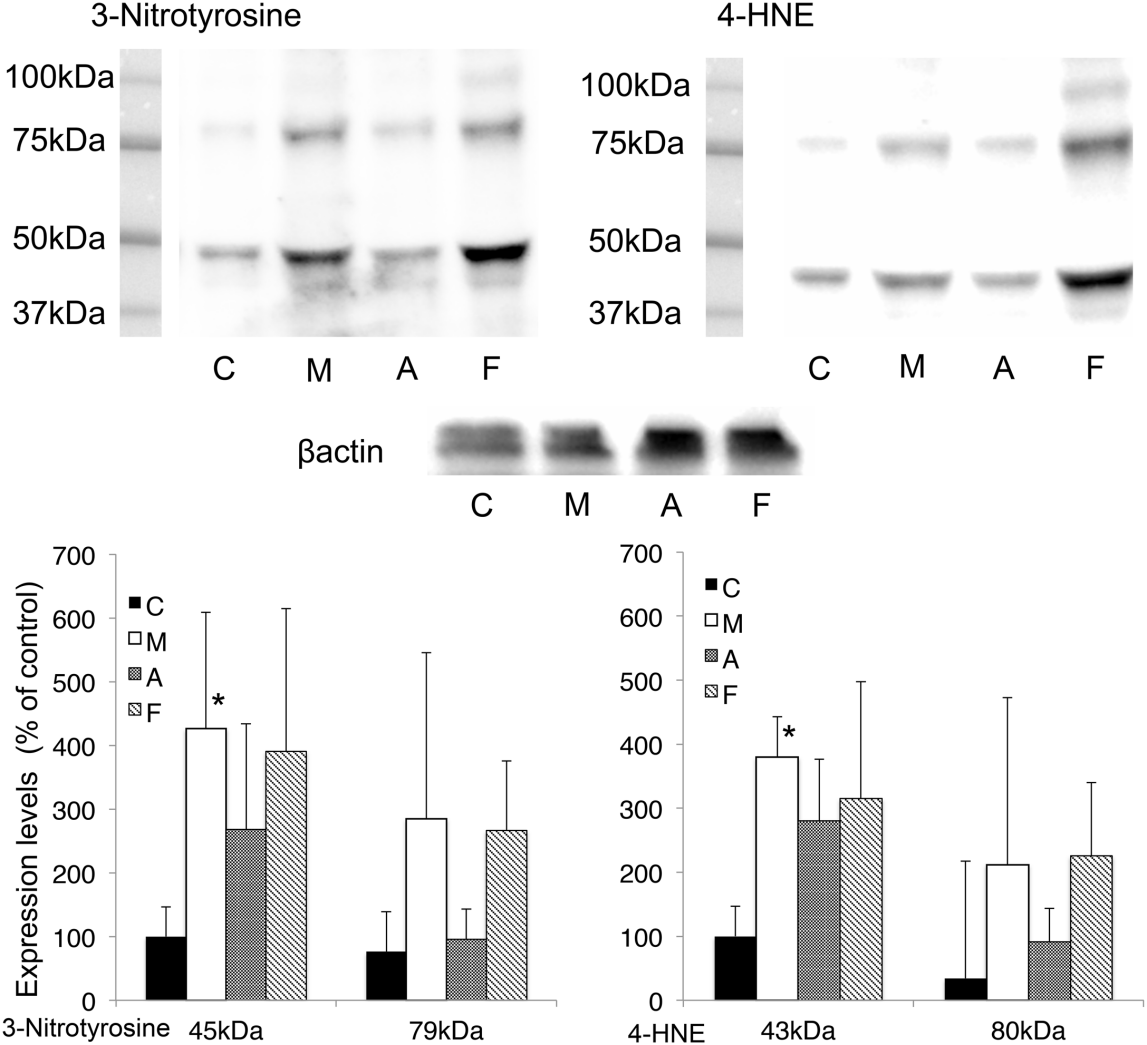
Effects of XOR inhibitors, showing changes in protein nitration and lipid peroxidation. Solid bars represent control groups (C). Open bars represent methylcellulose groups (M). Dotted bars represent allopurinol groups. Diagonal bars represent febuxostat groups (F). The intensity levels are given as the mean ± SD normalized to β-actin level for each sample. (n = 6) *P<0.05 vs. control. C; Control, M; Methylcellulose, A; Allopurinol, F; Febuxostat. The entire images are shown in S4–S6 Figs.

Fig 3 displays the effect of XOR inhibitors on the 3-nitrotyrosine- and 4-HNE-modified protein levels measured by means of western blotting in mouse samples taken 24 h after the 14-min ischemia reperfusion procedure. The intensities of the 3-nitrotyrosine and 4-HNE signals in the methylcellulose group were significantly higher than those in the control group (3-nitrotyrosine p = 0.037, 4-HNE p = 0.022). No significant differences in the levels of 3-nitrotyrosine- and 4-HNE-modified proteins were observed in the groups that received allopurinol and febuxostat as compared with those in the methylcellulose group.

### Effects of XOR inhibitors on oxidative stress-related gene expression levels in global brain I/R injury

Recent studies have provided evidence that ROS signaling pathways, such as the NF-κB signaling pathway, can cause cellular damage and death in cerebral I/R.[8] NF-κB is a proinflammatory transcription factor that is associated with I/R injury.[9] The activation of NF-κB is regulated at the posttranslational level, and it therefore cannot be monitored by the detection of new mRNA or protein.[10, 11] A recent study indicated that XO is involved in triggering IL-1β release, and blocking XO reduces IL-1β.[12]

Thus, we analyzed downstream target genes that are known to be regulated by NF-κB. These downstream inducible genes include cytokines (IL-1β and TNF-α), matrix metalloproteinase-9 (MMP-9) and intercellular adhesion molecules-1 (ICAM-1), which are known to be involved in neuronal injury, blood-brain barrier breakdown and inflammatory response after cerebral I/R injury.

**Fig 4:**
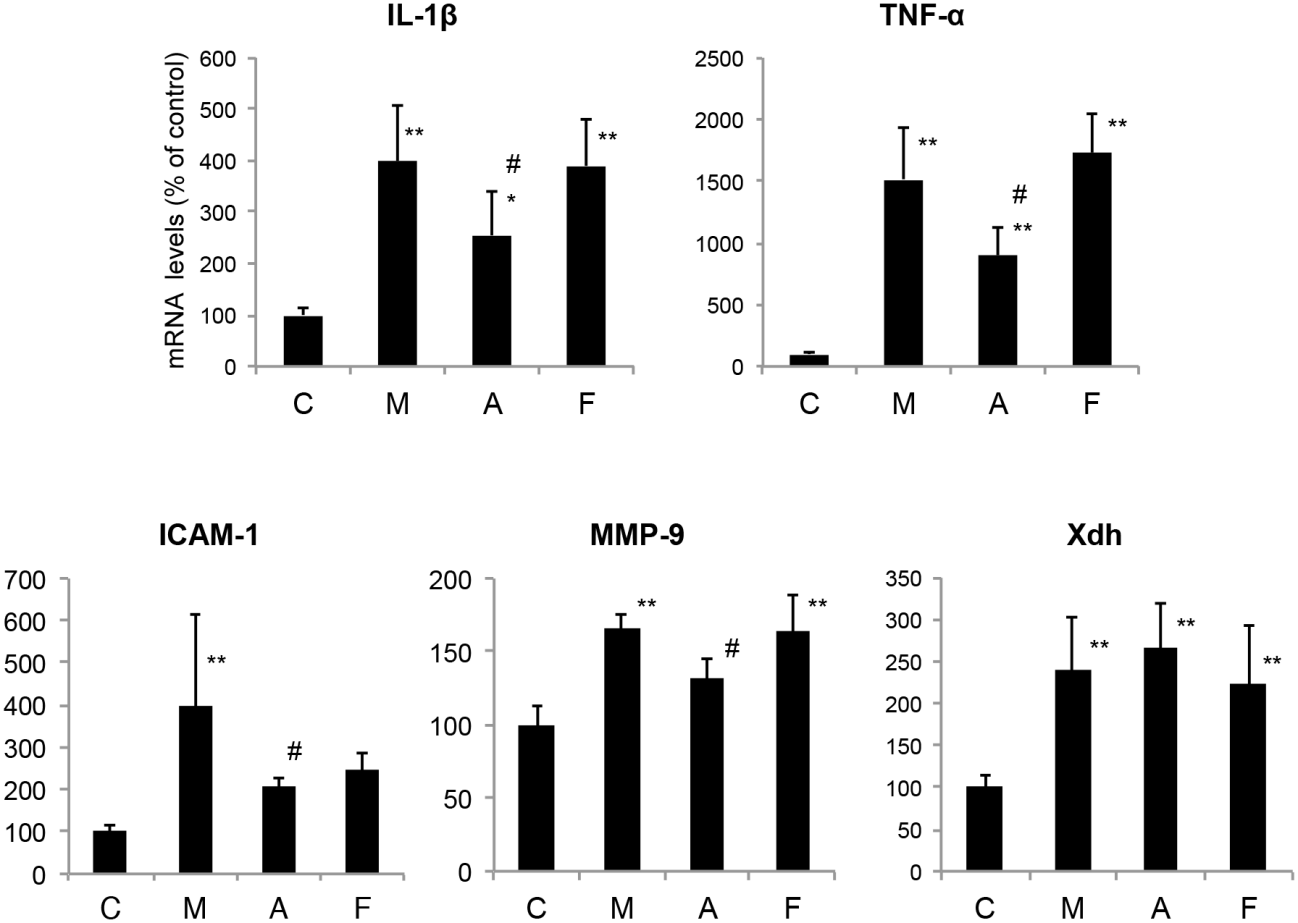
Effects of XOR inhibitors on gene expression after global brain I/R injury. The mRNA levels are given as the mean ± SD normalized to β-actin level for each sample. (n = 7)*P<0.05, **P<0.01 vs. control. #P<0.05 vs. methylcellulose. IL1β; interleukin-1β, TNF-α; tumor necrosis factor-α, ICAM-1; intercellular adhesion molecule-1, MMP-9; matrix metalloproteinase-9, Xdh; xanthine dehydrogenase, C; Control, M; Methylcellulose, A; Allopurinol, F; Febuxostat.

Fig 4 showed comparisons of the mRNA levels. All mRNA levels were significantly higher in the injured groups than in the uninjured group. The administration of allopurinol resulted in a statistically significant decrease in IL-1β, TNF-α, ICAM-1, MMP-9 mRNA expression compared with the methylcellulose group. However, the febuxostat group showed similar IL-1β, TNF-α, ICAM-1, MMP-9 mRNA expression levels to the methylcellulose group. No difference in the upregulation of Xdh was detected when the XOR inhibitor groups were compared with the methylcellulose group.

## Discussion

The present study demonstrated that cerebral injury after 14 min of global brain ischemia followed by reperfusion in the 3-vessel occlusion model causes oxidative stress. Our previous report showed that allopurinol and febuxostat did not decrease brain I/R damage, as indicated by a histopathological evaluation of the CA1 and CA2 regions after global brain I/R injury.[6] In the present study, we further evaluated the neuroprotective effect of XOR inhibitors on the mouse 3-vessel occlusion model by analyzing oxidative stress markers.

Brain ischemia induces activation of the *N*-methyl-D-aspartate receptor, and the subsequent formation of superoxide and nitric oxide has been considered a direct signal for mitochondrial peroxynitrite anion generation. Peroxynitrite is one of the most potent ROS and is a marker of the reactive nitrogen species induced by nitric oxide synthase during episodes of oxidative stress. In particular, 3-nitrotyrosine is a tyrosine nitration product that is generated under pro-inflammatory conditions by reactive nitrogen.[13] The reaction of oxygen radicals with unsaturated fatty acids in lipids produces peroxides that give rise to α, β-unsaturated aldehydes, including malondialdehyde, 4-HNE and acrolein. These aldehydes covalently bind to proteins by reacting with thiol groups, and alter their function.[14] Therefore, we evaluated ROS production during global brain I/R injury by using 3-nitrotyrosine and 4-HNE as indicators. Proteins with 3-nitrotyrosine or 4-HNE modifications were detected by western blot and immunohistochemical analyses. In the western blot analysis, the levels of 3-nitrotyrosine and 4-HNE reached a maximum at 8 h after reperfusion, and these levels were sustained up to 96 h after reperfusion (Fig 1). Using immunohistochemical analysis, we confirmed that CA1 pyramidal neurons, CA2 pyramidal neurons, motor cortex neurons, and endocapillary cells showed staining for 3-nitrotyrosine and 4-HNE at 8 h after reperfusion following global brain ischemia. These results are consistent with previous reports. Cuzzocrea et al. reported that nitrotyrosine was markedly increased in hippocampal neurons at 4 days after reperfusion following 5-min forebrain ischemia in gerbils.[15] Yabuki and Fukunaga reported that CA1 pyramidal neurons showed immunohistochemical staining for 3-nitrotyrosine and 4-HNE and increased levels of 3-nitrotyrosine- and 4-HNE-immunoreactive proteins in western blot analysis at 11 days after a 20-min bilateral carotid artery occlusion.[16] In a transient focal cerebral ischemia model, a similar time course was reported for the expression levels of nitric oxide synthases, which were elevated from the 6-hour to the 24-hour time point and remained elevated at 7 days after reperfusion.[17, 18]

Furthermore, the expression levels of several oxidative stress-related genes were evaluated. RT-PCR analysis indicated that TNF-α, IL-1, ICAM-1, and MMP-9 mRNA levels were elevated (Fig 4) and suggested that NF-κB was activated by ROS produced during the 14-min global brain ischemia and 24-h reperfusion injury. We confirmed by means of immunohistochemical analyses that CA1 pyramidal neurons, CA2 pyramidal neurons, and motor cortex neurons showed staining for NF-κB at 8 h after reperfusion following global brain ischemia. NF-κB controls the expression of genes that encode pro-inflammatory cytokines (TNF-α, IL-1), cell surface molecules (ICAM-1), enzymes (MMP-9), and immune receptors.[19] The cytokines formed after ischemia stimulate the expression of adhesion molecules on endothelial cells and leukocytes, leading to leukocyte adhesion and extravasation into brain parenchyma.[20] However, no neutrophil infiltration was observed in the histochemical analysis in our study (Fig 2). It is possible that the time from reperfusion to sacrifice for specimen preparation was too short for neutrophil infiltration to occur.

Interestingly, allopurinol but not febuxostat had neuroprotective effects, although febuxostat has a higher bioavailability and a more potent XO-inhibitory effect than those of allopurinol (Fig 4).[21] Our RT-PCR study showed that the TNF-α, IL-1β, ICAM-1, and MMP-9 mRNA levels were reduced by the administration of allopurinol, but not by the administration of febuxostat (Fig 4). The properties of the two inhibitors are quite different; because of its greater XO selectivity, febuxostat does not cause the cross-inhibition that allopurinol exerts on other purine and pyrimidine metabolism enzymes, presumably because of its structural similarity to endogenous purines.[22] Moreover, febuxostat can be expected to cause prolonged enzyme inhibition *in vivo*, because the enzyme inhibitor complex is very stable and is not influenced by changes in the redox status of the cofactor.[23] These differences between the two XOR inhibitors may explain why only allopurinol could decrease the elevated expression of oxidative stress-related genes during I/R injury, even though it might be expected that febuxostat would be better able to inhibit oxidative stress caused by XOR.

Western blot analysis showed that febuxostat and allopurinol did not reduce the levels of 3-nitrotyrosine and 4-hydroxy-2-nonenal (4-HNE) produced in response to I/R injury (Fig 3), although we expected that both XOR inhibitors would have neuroprotective effects. XO is proposed to be a major ROS source in the brain, but this idea remains controversial. For example, in a permanent focal ischemia model in rats, Betz et al. found no correlation between enzyme inhibition with low doses of allopurinol and reduction in brain edema.[24] Lindsay et al. inhibited XO activity in rats by dietary depletion of the essential cofactor molybdenum and found no improvement in brain injury after focal cerebral ischemia and reperfusion.[25] XOR is not likely to be a major source of ROS production in our study, for the following reasons. It has been suggested that the upregulation of overall XOR activity as a consequence of ischemia and/or of substrate level changes could lead to increases in ROS.[26] Indeed, our RT-PCR results also suggested upregulation of XOR following I/R injury (Fig 4). However, the distribution of XOR in the brain is quite low,[27] though some in vivo studies found that XOR activity increases during hypoxia and reperfusion.[24, 25] On the other hand, we previously found that XOR expression levels in brain were extremely low compared with those in liver even after reperfusion.[6] These results are consistent with the finding that the effects of XOR inhibitors were limited in brain ischemia. Our RT-PCR results indicated that Xdh was upregulated approximately 2- to 3-fold in injured mice compared with uninjured mice; however, as the baseline XOR activity is so low, this does not necessarily imply that XOR is a major source of ROS production in ischemic brain.

Administration of allopurinol reduced the elevated expression levels of oxidative stress-related genes, and improved the neurological outcome, but there are possible explanations for this that do not involve XO-derived free radicals. Different from febuxostat, allopurinol is able to pass through the BBB,[28] and allopurinol may directly scavenge free radicals in the brain. Indeed, protective effects of allopurinol against various oxidative stresses have been reported.[29–33] It should be noted that at relatively high concentrations (>500 μM), allopurinol and oxypurinol are potent scavengers of hydroxyl radicals *in vitro*.[34] Betz et al. examined the effect of various doses of allopurinol on uric acid accumulation and brain edema formation. Though all doses of allopurinol greatly reduced the appearance of uric acid in the ischemic brain, only the highest dose of allopurinol had any beneficial effect on brain edema.[24]

In this study we observed that the administration of allopurinol reduced TNF-α, IL-1, ICAM-1, and MMP-9 mRNA levels, suggesting that allopurinol inhibited ROS-mediated NF-κB activation. As febuxostat had no effects on these markers, the reduced expression was considered to be due to a direct radical-scavenging effect of allopurinol. Western blot analysis indicated that administration of allopurinol has no effect of lipid peroxidation and tyrosine nitration. Allopurinol might be effective when the plasma oxypurinol level is sufficiently elevated to be effective for scavenging ROS.

3-Nitrotyrosine and 4-HNE were produced in the 3-vessel occlusion model in this study, and the levels of these compounds increased from 1 h to 8 h and remained elevated until 96 h. Thus, ROS were produced in the global brain I/R injury, not only in the hippocampal region, but also in the cortex region. However, XOR inhibitors did not reduce the levels of 3-nitrotyrosine- and 4-HNE-modified protein, while allopurinol reduced the mRNA levels of pro-inflammatory cytokines, cell surface molecules and enzymes. These results indicate that XO is most likely not an important source of ROS production in this model of global brain I/R injury, and further suggest that allopurinol exerts its brain tissue protective effect through its direct radical-scavenging activity.

Our findings have a number of potential clinical implications. Allopurinol and febuxostat suppress inflammatory reaction and, therefore should ameliorate diseases with an inflammation-linked pathogenesis, such as atherosclerosis.[35–38]. Recently, Ives et al. reported that XOR regulates macrophage IL1b secretion upon NLRP3 inflammasome activation, and febuxostat suppresses the activation by inhibiting XOR-generated ROS production.[12] Febuxostat has also been shown to reduce tissue damage in myocardial dysfunction and chronic kidney disease.[38] Thus, the mechanisms of the beneficial effects of XOR inhibitor treatment are likely to be multi-factorial.

Both allopurinol and febuxostat are potent XOR inhibitors, but their brain protective effects are different. The reason for the difference is likely to be differences in their chemical properties, not in their XOR-inhibitory effects. Allopurinol is BBB-permeable and has a potent radical-scavenging character. Thus, it would be effectively transferred to the damaged brain and reduce ROS levels there. On the other hand, febuxostat is not BBB-permeable and does not have radical-scavenging ability. Thus, it is important to take into account the overall chemical properties of XOR inhibitors in assessing their tissue-protecting ability.

## Supporting Information

S1 Fig Uncropped blot of 3-nitrotyrosine (Fig 1). The protein indicated by the arrowhead was analyzed; other positive bands were not examined at this time.
(TIF)

S2 Fig Uncropped blot of 4-HNE (Fig 1). The protein indicated by the arrowhead was analyzed; other positive bands were not examined at this time.
(TIF)

S3 Fig Uncropped blot of β-actin (Fig 1). (TIF)

S4 Fig Uncropped blot of 3-nitrotyrosine (Fig 3). The protein indicated by the arrowhead was analyzed; other positive bands were not examined at this time. C; Control, M; Methylcellulose, A; Allopurinol, F; Febuxostat.
(TIF)

S5 Fig Uncropped blot of 4-HNE (Fig 3). The protein indicated by the arrowhead was analyzed; other positive bands were not examined at this time. C; Control, M; Methylcellulose, A; Allopurinol, F; Febuxostat.
(TIF)

S6 Fig Uncropped blot of β-actin (Fig 3). C; Control, M; Methylcellulose, A; Allopurinol, F; Febuxostat.
(TIF)

S7 Fig Inhibition of β-actin antibody with blocking peptide. Whole brain lysate (20 μg) was loaded on each lane and subjected to analysis. Two bands indicated by arrowheads were inhibited with blocking peptide. Lanes 1, 2 were without blocking peptide, and lanes 3, 4 were with blocking peptide.
(TIF)

S1 File Statistical result of Fig 1 (Table A), statistical result of Fig 3 (Table B) and statistical result of Fig 4 (Table C). (DOCX)
