# Supplementary figures and images for: The Effects of Xanthine Oxidoreductase Inhibitors on Oxidative Stress Markers following Global Brain Ischemia Reperfusion Injury in C57BL/6 Mice
Source: PLoS One. 2015 Jul 31;10(7):e0133980. doi: 10.1371/journal.pone.0133980 (PMC4521791; doi:10.1371/journal.pone.0133980)

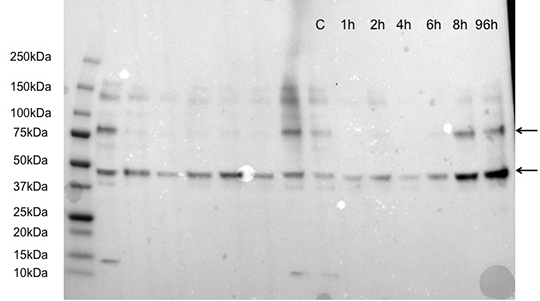

Supplement: S1 Fig — The protein indicated by the arrowhead was analyzed; other positive bands were not examined at this time. (TIF) [file pone.0133980.s001.tif]

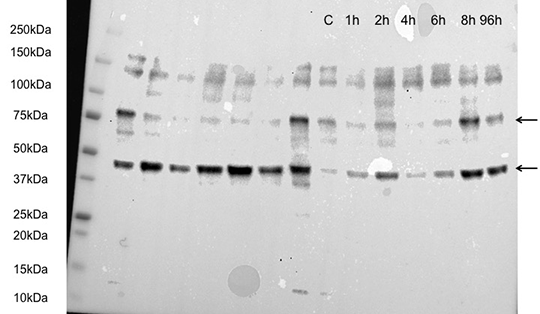

Supplement: S2 Fig — The protein indicated by the arrowhead was analyzed; other positive bands were not examined at this time. (TIF) [file pone.0133980.s002.tif]

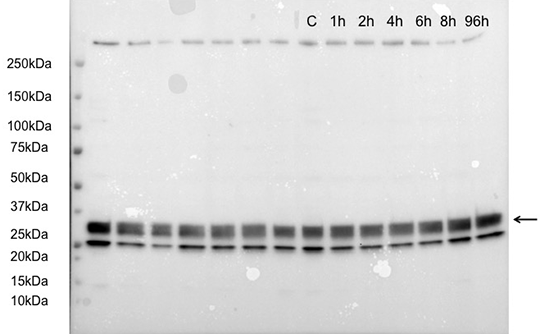

Supplement: S3 Fig — (TIF) [file pone.0133980.s003.tif]

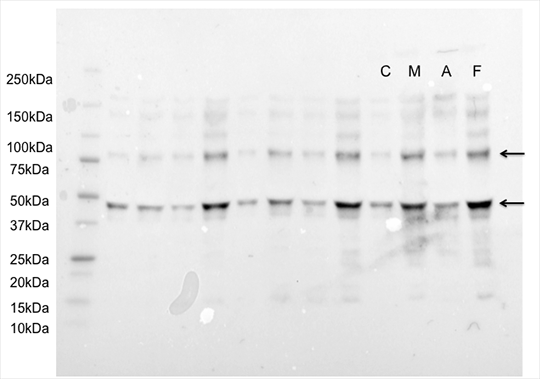

Supplement: S4 Fig — The protein indicated by the arrowhead was analyzed; other positive bands were not examined at this time. C; Control, M; Methylcellulose, A; Allopurinol, F; Febuxostat. (TIF) [file pone.0133980.s004.tif]

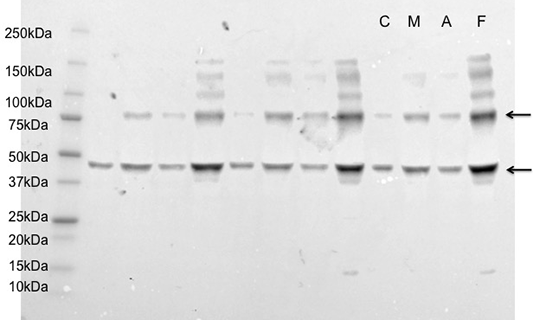

Supplement: S5 Fig — The protein indicated by the arrowhead was analyzed; other positive bands were not examined at this time. C; Control, M; Methylcellulose, A; Allopurinol, F; Febuxostat. (TIF) [file pone.0133980.s005.tif]

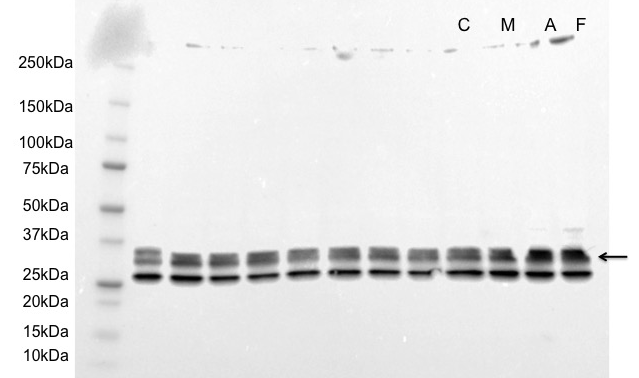

Supplement: S6 Fig — C; Control, M; Methylcellulose, A; Allopurinol, F; Febuxostat. (TIF) [file pone.0133980.s006.tif]

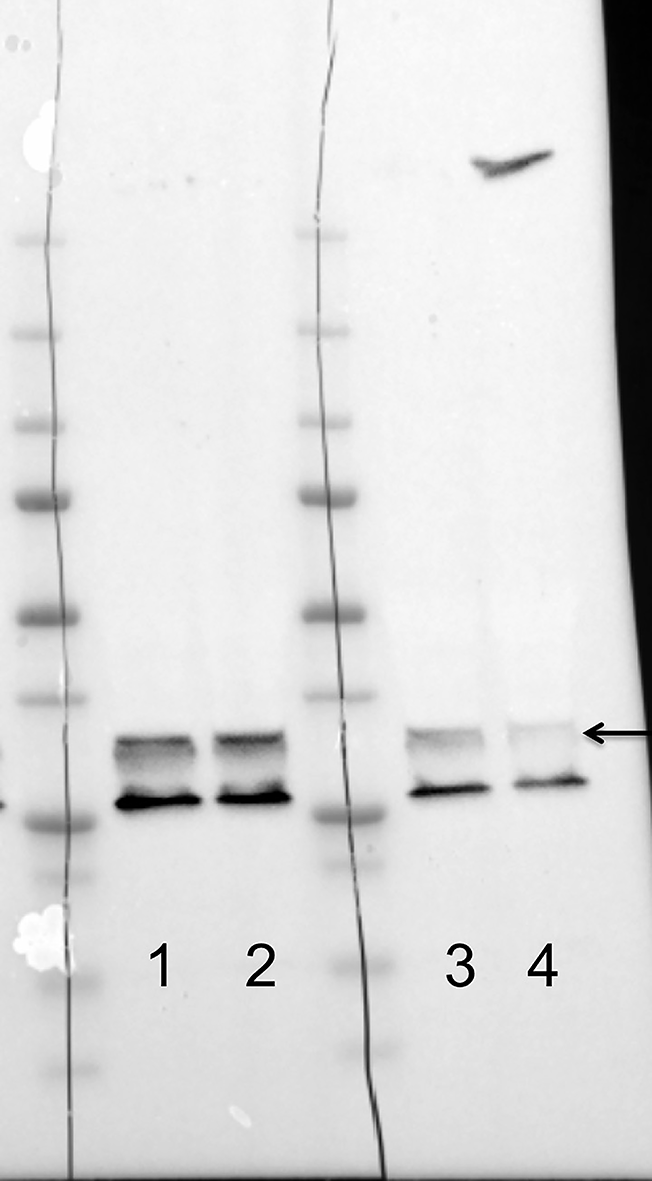

Supplement: S7 Fig — Whole brain lysate (20 μg) was loaded on each lane and subjected to analysis. Two bands indicated by arrowheads were inhibited with blocking peptide. Lanes 1, 2 were without blocking peptide, and lanes 3, 4 were with blocking peptide. (TIF) [file pone.0133980.s007.tif]
