# Supplementary material for: The Effects of Xanthine Oxidoreductase Inhibitors on Oxidative Stress Markers following Global Brain Ischemia Reperfusion Injury in C57BL/6 Mice
Source: PLoS One. 2015 Jul 31;10(7):e0133980. doi: 10.1371/journal.pone.0133980 (PMC4521791; doi:10.1371/journal.pone.0133980)
Supplement: S1 File — (DOCX) [file pone.0133980.s008.docx]

**TABLE A**

|  | 3-Nitrotyrsoine　45kDa (%) | p Value* | 3-Nitrotyrsoine 79kDa (%) | p Value* | 4-HNE  43kDa (%) | p Value* | 4-HNE  80kDa (%) | p Value* |
| --- | --- | --- | --- | --- | --- | --- | --- | --- |
| Control | 100 ± 60.54 | - | 34.5 ± 18.97 | - | 100 ± 57.67 | - | 37.93 ± 31.63 | - |
| 1h | 168.94 ± 78.93 | 0.148 | 57.79 ± 47.59 | 1.000 | 190.84 ± 99.19 | 1.000 | 37.78 ± 22.39 | 1.000 |
| 2h | 185.56 ± 87.15 | 1.000 | 73.11 ± 40.72 | 0.690 | 206.23 ± 80.50 | 1.000 | 53.73 ± 25.17 | 1.000 |
| 4h | 178.77 ± 89.79 | 1.000 | 111.58 ± 123.70 | 1.000 | 204.57 ± 92.19 | 1.000 | 90.86 ± 63.90 | 1.000 |
| 6h | 248.71 ± 85.56 | 0.614 | 114.86 ± 82.47 | 0.996 | 271.10 ± 106.44 | 0.128 | 93.63 ± 24.97 | 1.000 |
| 8h | 403.66 ± 126.91 | 0.032* | 227.48 ± 148.38 | 0.542 | 308.83 ± 142.16 | 0.033* | 161.72 ± 52.70 | 1.000 |
| 96h | 376.08 ± 159.03 | 0.048* | 200.47 ± 186.14 | 1.000 | 294.69 ± 127.53 | 0.049* | 181.14 ± 135.38 | 0.730 |

* The p values were calculated using the repeated-measures ANOVA with a Greenhouse-Geisser correction followed by post hoc tests using the Bonferroni correction. *p<0.05 vs. control

**TABLE B**

|  | Control (%) | Methylcellulose (%) | p Value* | Allopurinol (%) | p Value* | Febuxostat (%) | p Value* |
| --- | --- | --- | --- | --- | --- | --- | --- |
| 3-Nitrotyrosine 45kDa | 100 ± 46.46 | 426.1 ± 182.88 | (0.037*) | 268.13 ± 165.53 | 0.919 (0.778) | 390.72 ± 224.01 | 1.000 (0.077) |
| 3-Nitrotyrosine 79kDa | 76.22 ± 63.20 | 284.73 ± 260.80 | (0.217) | 95.46 ± 47.71 | 0.329 (1.000) | 266.22 ± 108.99 | 1.000 (0.324) |
| 4-HNE 43kDa | 100 ± 31.19 | 379.49 ± 169.64 | (0.022*) | 280.08 ± 96.80 | 0.284 (0.284) | 314.81 ± 182.96 | 1.000 (0.122) |
| 4-HNE 80kDa | 34.2 ± 29.33 | 211.9 ± 176.98 | (0.112) | 91.1 ± 52.78 | 0.582 (1.000) | 225.47 ± 114.67 | 1.000 (0.073) |

* The p values were calculated using the one-way ANOVA. *p<0.05 vs. Methylcellulose (*p<0.05 vs. control)

**TABLE C**

|  | Control (%) | Methylcellulose (%) | p Value* | Allopurinol (%) | p Value* | Febuxostat (%) | p Value* |
| --- | --- | --- | --- | --- | --- | --- | --- |
| IL1β | 100 ± 14.39 | 397.31 ± 110.16 | (<0.001*) | 254.82 ± 86.42 | 0.045* (0.020*) | 389.82 ± 90.12 | 1.000(<0.001*) |
| TNFα | 100 ± 16.42 | 1516.23 ± 411.31 | (<0.001*) | 899.38 ± 224.71 | 0.019* (0.001*) | 1720.83 ± 318.47 | 0.626(<0.001*) |
| ICAM1 | 100 ± 15.04 | 397.35 ± 216.8 | (<0.001*) | 208.82 ± 21.41 | 0.042* (0.611) | 249.69 ± 37.72 | 0.180 (0.168) |
| MMP9 | 100 ± 13.30 | 165.11 ± 11.26 | (<0.001*) | 131.04 ± 13.99 | 0.028* (0.054) | 163.99 ± 25.49 | 1.000 (<0.001*) |
| Xdh | 100 ± 14.014 | 239.98 ± 63.03 | (<0.001*) | 268.27 ± 50.00 | 1.000(<0.001) | 224.34 ± 67.64 | 1.000(<0.001*) |

* The p values were calculated using the one-way ANOVA. *p<0.05 vs. Methylcellulose (*p<0.05 vs. control)
